# Extraction of bioactive compounds from *Psidium guajava* leaves and its utilization in preparation of jellies

**DOI:** 10.1186/s13568-021-01194-9

**Published:** 2021-03-01

**Authors:** N. S. Sampath Kumar, Norizah Mhd Sarbon, Sandeep Singh Rana, Anjani Devi Chintagunta, S. Prathibha, Satheesh Kumar Ingilala, S. P. Jeevan Kumar, B. Sai Anvesh, Vijaya Ramu Dirisala

**Affiliations:** 1 Department of Biotechnology, Vignan’s Foundation for Science, Technology and Research, Vadlamudi, Andhra Pradesh 522213 India; 2 Faculty of Fisheries and Food Science, Universiti Malaysia Terengganu, 21030 Kuala Terengganu, Terengganu Malaysia; 3 Division of Food Technology, Department of Chemical Engineering, Vignan’s Foundation for Science, Technology and Research, Vadlamudi, Andhra Pradesh 522213 India; 4 ICAR-Directorate of Floricultural Research, Pune, Maharashtra 411005 India; 5 Department of Chemistry, Presidency College, Chennai, 600005 India

**Keywords:** Antioxidant activity, Antimicrobial activity, Bioactive compounds, Guava leaf extract, Jelly, Texture analysis

## Abstract

*Psidium guajava* L. (guava) is predominantly grown throughout the world and known for its medicinal properties in treating various diseases and disorders. The present work focuses on aqueous extraction of bioactive compounds from the guava leaf and its utilization in the formulation of jelly to improve the public health. The guava leaf extract has been used in the preparation of jelly with pectin (1.5 g), sugar (28 g) and lemon juice (2 mL). The prepared guava leaf extract jelly (GJ) and the control jelly (CJ, without extract) were subjected to proximate, nutritional and textural analyses besides determination of antioxidant and antimicrobial activities. GJ was found to contain carbohydrate (45.78 g/100 g), protein (3.0 g/100 g), vitamin C (6.15 mg/100 g), vitamin B3 (2.90 mg/100 g) and energy (120.6 kcal). Further, the texture analysis of CJ and GJ indicated that both the jellies showed similar properties emphasizing that the addition of guava leaf extract does not bring any change in the texture properties of jelly. GJ exhibited antimicrobial activity against various bacteria ranging from 11.4 to 13.6 mm. Similarly, GJ showed antioxidant activity of 42.38% against DPPH radical and 33.45% against hydroxyl radical. Mass spectroscopic analysis of aqueous extract confirmed the presence of esculin, quercetin, gallocatechin, 3-sinapoylquinic acid, gallic acid, citric acid and ellagic acid which are responsible for antioxidant and antimicrobial properties.
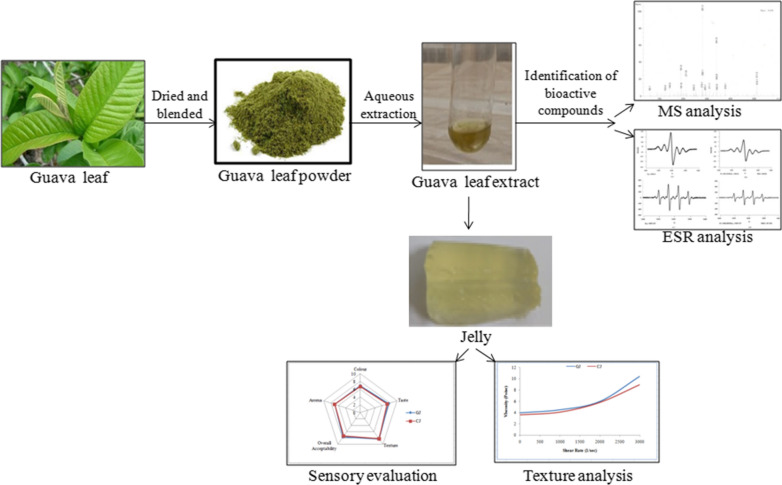

## Introduction

*Psidium guajava* L. (guava) grows predominantly in various countries with in tropical and subtropical regions. Guava has several medicinal properties and is well known for treating dysentery, vertigo, skin problems, jaundice, cerebral ailments, diabetes etc. It is rich in phenolics, flavonoids, triterpenoids, tannins, vitamins, essential oils and sesquiterpene alcohols (Altemimi et al. 2017; Singh et al. 2019). Guava leaves mainly constitute rutin, naringenin, gallic acid, catechin, epicatechin, kaempferol, isoflavonoids, and flavonoids such as quercetin and guaijaverin that are well known for their antimicrobial, antioxidant and anti-inflammatory actions (Gutiérrez-Grijalva et al. 2018; Shaheena et al. 2019). Due to the copious benefits, guava is cultivated worldwide where, India stands in the first position with the production of 40,54,000 million tons (MT) from 2,65,000 hectares (ha) in the year 2017–18 (Horticultural Statistics at a Glance 2018).

However, due to inappropriate transportation, handling and processing, the fruit wastage is nearly 20–25% of total guava production which was reduced by processing into various value added products viz., juice, jam, jelly, wine and toffee (Kumari et al. 2017; Jacob et al. 2016). Jellies attract the attention of consumers due to their colour, smooth texture, flavour and sweet taste. Generally, jelly is prepared by boiling a clear fruit extract with sugar, citric acid, additives and pectin to give a semi-solid appearance. The synthetic additives for colour and flavour are considered as potential carcinogenic or neurotoxic agents and hence, their usage was restricted. Concomitantly, the demand for clean label foods with natural ingredients rich in antioxidant properties has attracted the attention of consumers and food industries (Singh et al. 2019).

Keeping in view of food producer’s requirements, the present work is aimed at the preparation of jellies rich in bioactive compounds. Despite of using a well studied guava fruit as an ingredient in the jelly preparation, a relatively less exploited guava leaves were employed in the present study. The aqueous extraction was performed to isolate the bioactive compounds from the guava leaves and their characterization has been carried out by Mass spectroscopy. The jellies with and without guava extract were prepared by following standard protocol and their proximate, texture and organoleptic analysis were performed to achieve the objective. The jelly prepared with guava leaf extract was considered as vehicle to promote the intake of bioactive compounds rich in antioxidant and antibacterial properties.

## Materials and methods

All chemicals used for different experimental studies such as chloroform, conc.H_2_SO_4_, ammonium hydroxide, methanol, formic acid, acetonitrile, ampicillin, glucose, phenol red, 5,5-dimethylpyrroline-*N*-oxide (DMPO), FeSO_4_, H_2_O_2_ were of analytical grade procured from M/s. SD Fine, M/s. Qualigens and M/s. SRL, India. Pectin was procured from Purix India Pvt. Ltd., 2,2-diphenyl-1-picrylhydrazyl (DPPH) and Muller Hinton broth were procured from M/s. Sigma and M/s. HiMedia respectively.

### Sample collection and processing

Fresh leaves of *P. guajava* L. (guava) were collected from the premises of Vignan’s Foundation for Science, Technology and Research, Vadlamudi (16.2334° N, 80.5509° E). The leaves were rinsed gently with double distilled water, shade dried and powdered using a blender. The powder was then passed through aluminium sieve (1 mm) to get uniform particle size. Guava leaf powder was stored in an air tight container for further studies.

### Preparation of guava leaf extract

The guava leaf powder (20 g) was boiled at 90 °C in 100 mL of double distilled water in sterile Erlenmeyer flask (150 mL) for 30 min. The mixture was centrifuged at 4000 rpm for 10 min (Biswas et al. 2013). The supernatant was separated and stored at 4 °C for further studies.

### Phytochemical screening of guava leaf extract

The guava leaf extract was tested for the presence of bioactive components. A mixture containing glacial acetic acid (2 mL), 2 drops of FeCl_3_ and 2 mL of conc. H_2_SO_4_ was mixed with aqueous extract. A brown ring at the interface confirms the presence of glycosides. For detection of saponin, the extract was taken in a measuring cylinder, diluted with distilled water and was shaken vigorously for the formation of foam (Kokate 1999). The presence of tannins and phenols were confirmed by formation of blue-green/black colour upon mixing the extract (1 mL) with 2 mL of FeCl_3_ (2%, w/v) (Evans 1997). For screening terpenoids, 5 mL of extract was mixed with 2 mL of chloroform and 3 mL of conc. H_2_SO_4_ to form a layer. The presence of terpenoids is confirmed by the formation of reddish brown colour at the interface. Besides, the presence of flavonoids is confirmed by yellow fluorescence upon treating 0.5 mL of extract with 5 mL of 10% ammonium hydroxide solution (Evans 1997).

### Characterization of phytochemicals by mass spectroscopy

Mass Spectroscopy is an analytical technique used for separation and identification of various components present in a mixture. Agilent 1100 LC/MS System with Chemstation Rev.A.09.01 (1206) software was used for sample processing and analysis. The extract was mixed in methanol in 1:10 ratio and 20 µL of the sample was directly injected into the mobile phase [0.1% formic acid in water (50%) and acetonitrile (50%)] at a flow rate of 0.5 mL/min. The electrospray ionization (ESI) was set in negative ionization mode in 60–200 V and capillary voltage at 4000 V. Nitrogen is used as nebulizing gas at 350 °C and 30 psi pressure with flow rate of 8–10 L/min.

### Preparation of jelly and its proximate analysis

Two types of jellies, with and without guava leaf extract was prepared by mixing pectin, sugar and lime juice as the major ingredients. In 100 mL of water, pectin (1.5 g) along with 28 g of sugar and guava leaf extract (10%, v/v) was mixed and boiled at 100 °C till the mixture was thickened. To minimize pre-gelling and hydrolysis of pectin, 2 mL of *Citrus aurantifolia* was added at the end of boiling. The mixture was cooled for the jelly formation and stored in airtight container. Further, the jellies were subjected to proximate composition (AOAC 2008) and texture analysis.

### Physicochemical analysis

The total soluble solids (TSS) of samples is determined by using hand refractometer (Model- MCP Metal and PP), having range of 0–100^o^Brix (Ghosh et al. 2017). The digital pH meter (Mettler Toledo, USA) was used to measure the pH of the samples.Viscosity of the samples was determined by Brookfield Viscometer (Model DV1 Digital Brookfield, Middleboro, USA) at 30 ± 0.5 °C (Keshani et al. 2012).

### Texture analysis

Texture profile of jellies was determined by using CT3 texture analyser connected to a cylindrical probe (TA4/1000, 20 mm L) at pre-test speed: 2.00 mm/s, test speed: 1.00 mm/s, post-test speed: 1.00 mm/s and load cell: 10,000 g. The total profile analysis of jellies was performed for two cycles for 5 replications. Various properties such as firmness, cohesiveness, chewiness, springiness and gumminess were determined by calibrated load cell through measuring the resistance of material against force applied by the spindle, and analyzed results were taken from the installed Texture Pro CT Software in their respective units (Ghosh et al. 2017).

### Determination of antibacterial activity

Guava leaf extract was assessed for antibacterial activity against *Proteus vulgaris* (MTCC 744), *Streptococcus mutans* (MTCC 890), *Bacillus subtilis* (MTCC 1305) and *Staphylococcus aureus* (MTCC 9760) by disc diffusion method. Each strain was maintained on nutrient agar slant at 37 °C for 24 h. The inoculum was uniformly spread over the surface of the nutrient agar plate and allowed to dry. The sterile disc (6 mm) was loaded with 60 µL of guava leaf extract and placed on the inoculated agar plate before incubating at 37 °C. The zone of inhibition obtained due to antagonistic effect of guava aqueous leaf extracts was recorded after 24 h of incubation. The double distilled water used for extraction and Ampicillin (50 mg/mL) was considered as negative and positive controls. The diameter of inhibitory zone was measured both vertically and horizontally and its average (in mm) was considered (Guntur et al. 2018). Similarly, antibacterial activity of jelly prepared with and without guava leaf extract was also determined.

### Determination of antioxidant activity

Guava leaf extract (50 µg/µL) was thoroughly dissolved in DMPO (0.5 M, 20 µL) mixed with FeSO_4_ (15 mM, 20 µL) and H_2_O_2_ (15 mM, 20 µL) in a phosphate buffer solution (pH 7.4), and filled into a quartz tube. Radical concentration was quantified after 120 s in an electron spin resonance (ESR) spectrometer (Bruker, Germany). The experimental conditions employed were power 5 mW; magnetic field 336.5 ± 5 mT; amplitude 1 × 1000; modulation frequency 9.41 GHz; sweep time 30 s (Nazeer et al. 2013). In similar fashion, an aliquot of 50 µg/µL guava leaf extract was added to 50 µL of DPPH dissolved in ethanol at 60 µM concentration and tested. Similar parameters were deployed for assessment of antioxidant activity of jelly prepared with and without guava leaf extract. Radical scavenging potentiality was calculated with the following equation:

$$\text{Radical}\; \text{scavenging}\; \text{ability} \left({\%}\right)= \frac{({\text{H}}_{0}-\text{H})}{{\text{H}}_{0}} \times 100,$$

where H indicates relative peak height of radical signals with sample (guava leaf aqueous extract/jelly). H_0_ indicates relative peak height of radical signals without sample (guava leaf aqueous extract/jelly). Guava leaf extract from 25 to 125 µg/µL was evaluated to determine 50% of inhibition concentration (IC50) of DPPH radical.

### Sensory evaluation

Sensory evaluation of the jelly samples were conducted through a panel of 20 members (8 female, 12 males, of age group 20–40) using a hedonic scale ranging from least preferred (1) to most preferred (9) (Watts et al. 1989). The products were served to all the members for examining the quality and assign score for the characteristics viz., color, taste, texture, aroma and overall acceptability. Drinking water was offered to the panelists to cleanse their pallet after tasting each jelly sample. A questionnaire was supplied to each panelist for assigning their scores (Reddy et al. 2015).

### Statistical analyses

All the experiments were conducted on three replicates and data was expressed as mean ± standard deviation. The statistical analysis was performed using IBM SPSS statistics for windows, 20.0 software (IBM Corp., Armonk, N.Y., USA).

## Results

### Extraction and characterization of bioactive compounds

Guava leaf is known for possessing good source of bioactive compounds and in order to utilize these compounds, aqueous extract was prepared and assessed for qualitative confirmation of secondary metabolites. As shown in Table 1, glycosides, flavonoids, saponins, phenols, terpenoids and tannins were present in the extract. Further, specific confirmation and characterization of secondary metabolites present in the extract was carried out using mass spectroscopy (MS) based on the mass-to-charge ratio of ions.

**Table 1:** Phytochemical analysis of guava leaf extracts

Compound	Glycosides	Flavonoids	Saponins	Phenols	Tannins	Terpenoids
*Psidium guajava L.* (guava) etract	+	+	+	+	+	+

+: present

**Fig. 1:**
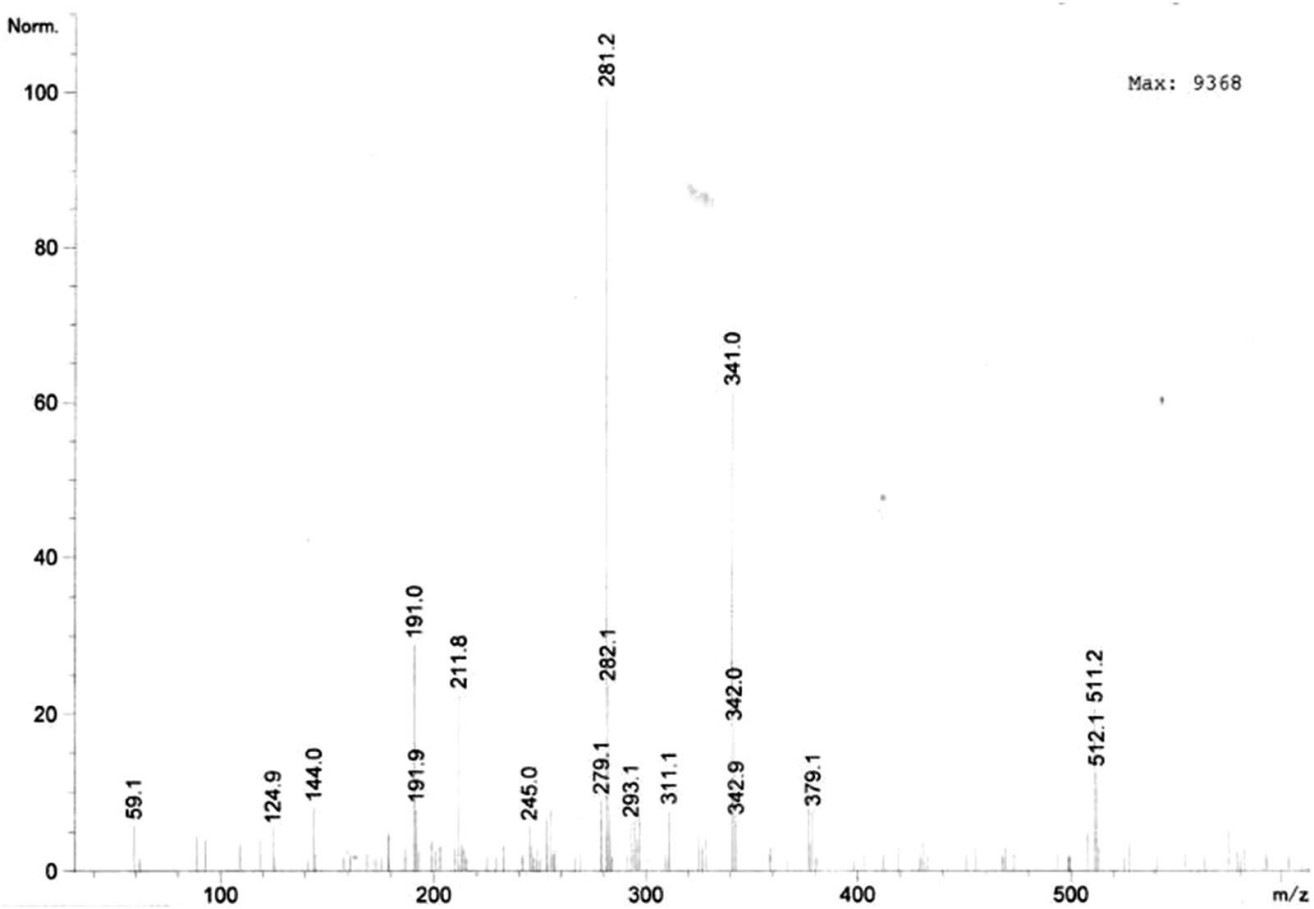
Liquid chromatography mass spectrometry (LC–MS) analysis of guava leaf extract

Upon subjecting the extracted compounds to negative mode mass spectroscopy several peaks were observed (Fig. 1). The peaks at m/z 281.2 and 293.1 indicate the presence of fragments of esculin (C_17_H_26_O_4_) and quercetin (C_15_H_10_O_7_). Further, the peak at m/z 311.1 indicates the presence of gallocatechin (C_15_H_14_O_7_) in the guava extract. Moreover, the peak at m/z 379.1 represents the compounds 3-sinapoylquinic acid (C_17_H_20_O_9_). Apart from these compounds, peaks at m/z 144.0, 191.0 and 341.0 represent the presence of gallic acid (C_7_H_6_O_5_), citric acid (C_6_H_8_O_7_) and ellagic acid (C_14_H_6_O_8_) respectively (Table 2).

**Table 2:** Compounds identified by mass spectroscopy and their properties

Compound	Peak (m/z)	Class	Chemical name/structure	Reported biological, activities	References
Quercetin	293.1	Flavonoid	3,3′,4′,5,7-Pentahydroxy flavone	Antioxidant, anti-inflammatory and anti-allergy	Sharma and Gupta (2010)
Gallocatechin	311.1	Flavonoid	Flavan-3,3′,4′,5,5′,7-hexol	Antioxidant and anti-cancer	Legeay et al. (2015)
Esculin	281.2	Hydroxycoumarin	6-*O*-Beta-d-glucoside of esculetin	Antioxidant, anticancer and a metabolite	Wang et al. (2013)
3-Sinapoylquinic acid	379.1	Quinic acid derivative	Cyclohexane ring with four hydroxyl groups at positions 1, 3.4, and 5, and carboxylic acid at position 1	Antioxidant and anti-cancer	Legeay et al. (2015)
Ellagic acid	341.0	Phenolic acid	Dilactone of hexahydroxydiphenic acid	Antioxidant, antihepatotoxic, antisteatosic, anticholestatic, antifibrogenic, anti-hepatocarcinogenic, antibacterial, and antiviral	García-Nino and Zazueta (2015)
Gallic acid	144.0	Phenolic acid	Trihydroxybenzoic acid	Antibacterial, anti-fungal, antiviral, anti-inflammatory, antioxidant, anticancer, anti-diabetic	Nayeem et al. (2016)
Citric acid	191.0	Carboxylic acid	2-Hydroxy-1,2,3-propanetricar-boxylic acid	Antibacterial,calculi dissolution agent, anticoagulant, preservative agent	García-Nino and Zazueta (2015)

### Proximate and vitamin composition of jellies

Upon considering the demand for value added products and fortified foods, bioactive compounds extracted from guava leaf has been used in the preparation of jelly referred as guava leaf extract jelly (GJ). The proximate analysis of GJ was carried out according to the standard protocols with reference to the control jelly (CJ) which was prepared without the addition of the extract. The carbohydrate content (g/100 g) of the two jellies was found to be similar as equal quantity of sugar has been added to the jellies (Table 3). Besides, the energy of GJ was found to be slightly higher (120.6 ± 1.20 kcal) than that of CJ (105.6 ± 1.50 kcal). There was no significant difference in the protein (2.8 ± 0.25 and 3.0 ± 0.19), fat (0.16 ± 0.02 and 0.20 ± 0.04 g/100 g) and vitamin contents in both the jellies.

**Table 3:** Proximate and nutritional analyses of jellies

Nutrition composition	CJ (100 g)	GJ (100 g)
pH	3.72 ± 0.04	3.50 ± 0.02
Moisture (%)	44.25 ± 0.33	45.05 ± 0.25
Dry matter (%)	55.02 ± 0.40	54.00 ± 0.25
Ash (%)	0.50 ± 0.10	0.59 ± 0.20
Energy (kcal)	105.6 ± 1.50	120.6 ± 1.20
Carbohydrate (g/100 g)	46.05 ± 0.25	45.78 ± 0.25
Protein (g/100 g)	2.8 ± 0.25	3.0 ± 0.19
Fat (g/100 g)	0.16 ± 0.02	0.20 ± 0.04
Vitamin C (mg/100 g)	6.24 ± 0.13	6.15 ± 0.20
Vitamin B3 (mg/100 g)	2.80 ± 0.35	2.90 ± 0.75

Data is expressed as mean ± SD

### Physicochemical analysis of jellies

**Fig. 2:**
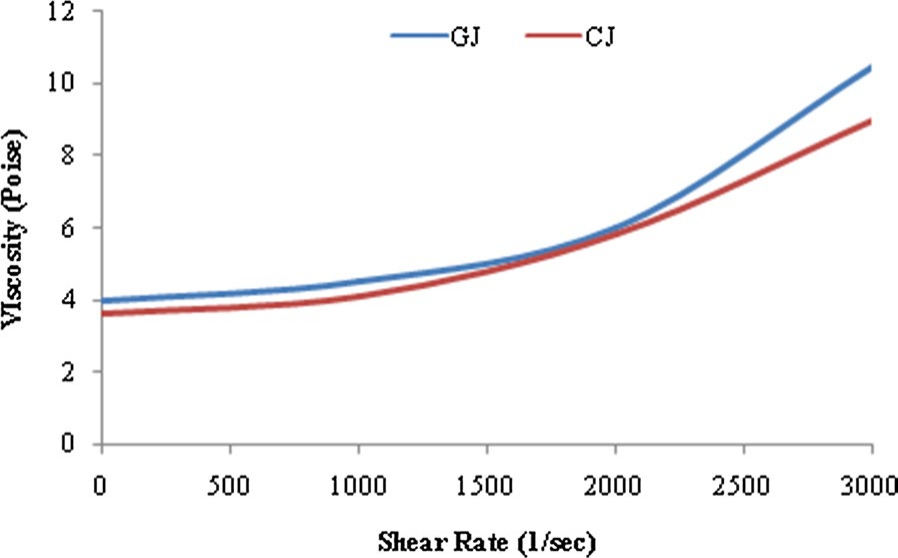
Viscosity of guava leaf extract jelly and control jelly as a function of shear rate

The soluble solids content, acidity and viscosity are the three main characteristics used for the evaluation of internal quality of the processed foods such as jellies and determinate their ultimate destination. The average TSS values for GJ and CJ were found to be 65.5^o^Brix and 65^o^Brix respectively. The jelly was found to be clear without the formation of lumps which infers that the straining process was done perfectly. Further, pH of the two jellies CJ and GJ was observed to be 3.72 ± 0.04 and 3.50 ± 0.02. The apparent viscosity of GJ was 286.45 Pa s, and the quality of jelly formed was high because of perfect gel formation, whereas for CJ apparent viscosity was 255.97 Pa s (Fig. 2).

### Texture analysis of jellies

The textural analysis of the prepared jellies GJ and CJ has been carried out by measuring their firmness, cohesiveness, chewiness, springiness and gumminess. The firmness of GJ and CJ was found to be 194 ± 12 g and 198 ± 10 g whereas, the cohesiveness value of GJ and CJ was 0.74 ± 0.04 and 0.76 ± 0.05 respectively. Besides, the chewiness of GJ and CJ was observed to be 15.0 ± 1.0 mJ and 15.5 ± 1.5 mJ, which are in close proximity to one another. Moreover, both the jellies GJ and CJ have exhibited nearly similar springiness i.e., 9.46 ± 0.5 mm and 9.04 ± 0.9 mm. Further, the gumminess of GJ (125 ± 9.6 g) is slightly higher than that of CJ (114 ± 5.2 g).

### Antibacterial activity of jellies

Antibacterial activity of the guava leaf extract and jellies were evaluated against *Bacillus subtilis, Proteus vulgaris, Staphylococcus aureus* and *Streptococcus mutans*. The highest and lowest inhibition zones formed due to antagonistic effect of guava leaf extract on *Bacillus subtilis* and *Staphylococcus aureus* was 14.1 ± 0.02 mm and 13.6 ± 0.01 mm respectively. Besides, the zone of inhibition of *Staphylococcus mutants* and *Proteus vulgaris* was observed to be 13.3 ± 0.01 mm and 12.1 ± 0.03 mm, respectively. Similar trend was observed with the jelly samples also (Table 4).

**Table 4:** Comparison of textural properties of guava leaf extract jelly (GJ) and control jelly (CJ)

Sample	Parameters				
	Firmness (g)	Cohesiveness	Chewiness (mJ)	Springiness (mm)	Gumminess (g)
GJ	194 ± 12	0.74 ± 0.04	15.0 ± 1.0	9.46 ± 0.5	125 ± 9.6
CJ	198 ± 10	0.76 ± 0.05	15.5 ± 1.5	9.04 ± 0.9	114 ± 5.2

Data is expressed as mean ± SD

### Antioxidant activity of jellies against DPPH and hydroxyl radical

Antioxidant activity of guava leaf extract and jellies (CJ and GJ) were tested against hydroxyl radical (*OH) and DPPH radical as shown in Table 4. Guava leaf extract was successful in scavenging DPPH* with 47.87% and 43.36% activity against *OH species. The IC50 value to guava leaf extract against DPPH radical was 62.7 µg/µL. Upon testing the jellies, GJ was found to scavenge both the free radicals with efficiency of 42.38% and 33.45% respectively. Thus, the results corroborate the antioxidant properties in the jelly.

### Sensory evaluation of jellies

**Fig. 3:**
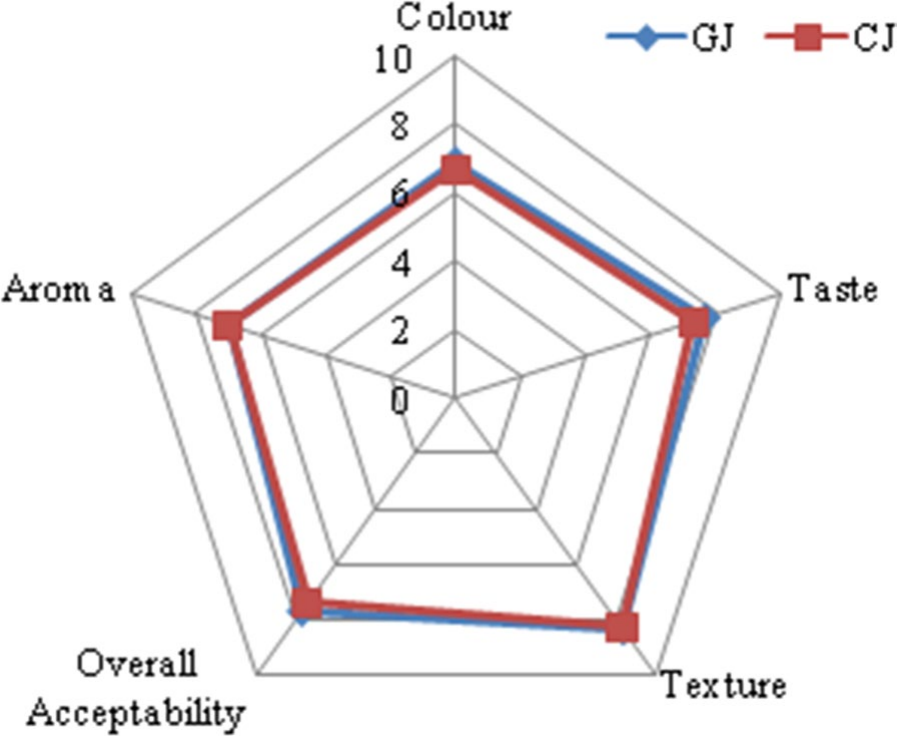
Sensory evaluations of control and guava leaf extract jellies

The sensory attributes viz., colour, texture, taste, aroma and overall acceptability of two jellies, GJ and CJ were evaluated by serving the jellies to panelists who have assigned scores following the hedonic scale from 1 to 9. Both the jellies were pale in colour with smooth texture. The score for colour of CJ and GJ was 6.7 ± 0.47 and 6.95 ± 0.69 respectively, whereas the score for taste was 7.3 ± 0.47 and 7.7 ± 0.86, respectively. CJ was sweet in taste and the GJ is moderately bitter and sour in taste. The score for texture CJ and GJ was 8.26 ± 0.64 and 8.35 ± 0.49, respectively while; the score for aroma was 7.0 for both the jellies. Besides, the overall acceptability was 7.42 ± 0.25 and 7.66 ± 0.47 respectively for CJ and GJ (Fig. 3). GJ is rich in bioactive compounds having anti-oxidant and antimicrobial activities.

## Discussion

The aqueous extract of guava leaves was screened for the presence of phytochemicals such as glycosides, flavonoids, saponins, polyphenols, terpenoids and tannins on qualitative basis. The extract was found to contain all the tested bioactive components (Table 1). Altemimi et al. (2017) also reported similarly about the presence of terpenoids, phenol and carbohydrates in the aqueous extract of guava leaves. The phytochemicals are found to possess various physiological activities, for instance, flavonoids are hydroxylated polyphenolic compounds produced against microbial infection in the plants in addition to antioxidant properties (Górniak et al. 2019). Similarly, saponins and tannins are found to possess inhibitory effect against certain gram positive bacteria that include *Staphylococcus aureus* and *Bacillus cereus* (Biswas et al. 2013).

Further, the guava leaf extract was subjected to negative mode mass spectroscopy that provides information about molecular structure of organic and inorganic compounds. Sugar and sugar alcohols, structure of glycosides, flavonoids, anthraquinones, aromatics, coumarin, isoflavonoids, cardenolides and triterpenes are generally detected using mass spectroscopy (Feil and Lun 2018). The mass spectrometry analysis of guava leaf extract confirms the presence of quercetin, gallocatechin, esculin, 3-sinapoylquinic acid, ellagic acid, gallic acid and citric acid that have several medicinal properties (Fig. 1; Table 2). Quercetin is involved in decreasing the mortality from heart disease besides exhibiting hypocholesterolemic and antioxidant activities (Sharma and Gupta 2010). Gallic acid inhibits pancreatic cholesterol esterase, which decreases cholesterol levels whereas, catechins are important as a preventive treatment for diabetes type 2 and obesity (Nayeem et al. 2016; Legeay et al. 2015). Ellagic acid prevents cancer and treats several viral and bacterial infections, while esculin acts as vasoprotective agent (Nayeem et al. 2016; Wang et al. 2013).

The bioactive compounds extracted from guava leaf have been used in the preparation of jelly referred as guava leaf extract jelly (GJ) and its proximate analysis was carried out in reference to the control jelly (CJ) which was prepared without guava leaf extract. The moisture, ash, carbohydrate, protein and vitamin C contents of GJ and CJ was found to be in the range of 44.25–45.05%, 0.50–0.59%, 45.78–46.05%, 2.8–3.0% and 6.15–6.24 mg/100 g respectively (Table 3). Similar trend was reported in case of red guava jams (Nissa et al. 2019). The moisture, ash, carbohydrate, protein and vitamin C contents in the jelly developed from varieties of guava were in the range of 33-38.5%, 0.08–0.19%, 61–66%, 0.02–0.04%, 7–11 mg/100 g (Joshi et al. 2017). Water-soluble vitamins (B and C) are not stored by the body since they are eliminated in urine and hence humans require their continuous supply in diet. Hence, adequate intake of these jellies will definitely help the consumer by lowering high cholesterol levels in the body and boost up their immunity.

Physicochemical analysis of the prepared jellies GJ and CJ was carried out to evaluate their internal quality. TSS explains the presence of high concentration of pectin which improves the solid holding capacity. The average TSS values for GJ and CJ were found to be 65.5^o^Brix and 65^o^Brix respectively, which were in the ideal range (65–70°Brix) for jelly (Sharma et al. 2011). Similarly, pH plays a prominent role in the gel firmness besides the pectin content in the jelly, because low pH increases the rigidity by enhancing the interactions between polymer to polymer than polymer to water (Seshadri et al. 2019). On the other hand, the higher pH contributes to the browning reaction during preparation and predominantly dissociates the carboxyl groups leading to alterations in the shape (Shinwari and Rao 2018).

Owing to semisolid nature, jellies exhibit both viscosity and elasticity properties and measure of these rheological properties are essential to assess the quality of the processed foods. The carbohydrates and acid which are used as ingredients in jelly preparation are responsible for enhancing the viscous nature besides improving the gel forming structure, consistency and the shelf life. The experimental analysis performed on GJ and CJ, showed that the viscosity decreases with rise in shear, which results in reorganization of the structure at larger shear forces. The apparent viscosity of GJ was 286.45 Pa s, and the quality of jelly formed was high because of perfect gel formation, where as for CJ apparent viscosity was 255.97 Pa s. As both the jellies have the same concentration of sugar and other ingredients except guava leaf extract in GJ, the viscosities of both the jellies are in close proximity with each other. Xiu et al. (2011) observed that different concentrations of the jellies exhibit different viscosities because of inter-molecular forces between molecules and water solute interactions. The jelly was of visco-elastic nature and from the available results it can be concluded that the GJ will follow the Herschel–Bulkley model of viscoplastic fluid (Fig. 2).

Texture analysis represents mastication operation and the parameters emphasized during the analysis are firmness, cohesiveness, chewiness, springiness and gumminess of jellies (Table 5). Concentrations of sugar and pectin have profound influence on the textural properties of prepared jellies (Royer et al. 2006). Besides sugar and pectin, pH also influences the firmness of the jelly which is prepared with 45% sugar. The firmness of GJ and CJ was found to be 194 ± 12 g and 198 ± 10 g respectively inferring no significant difference between the jellies. In addition, cohesiveness of product indicates rate of material deformation under mechanical enforcement. Cohesiveness is measure of the strength of internal structure and difficulty in breaking down the internal bonds (Hamedi et al. 2018). The cohesiveness value of GJ (0.74 ± 0.04), confirms that the jelly is easy to chew and swallow. Apart from the above mentioned properties, chewing is very important for the consumers’ acceptance of the product. It corresponds to the energy required to masticate a solid food into ready for swallowing state (Calvarro et al. 2016). The chewiness of GJ and CJ were observed to 15.0 ± 1.0 mJ and 15.5 ± 1.5 mJ respectively which are in close proximity to one another.

**Table 5:** Antibacterial and antioxidant activity of guava leaf extract and jellies

Samples	Antibacterial activity—zone of inhibition (mm)				Antioxidant activity (%)	
	*P. vulgaris*	*S. mutans*	*B. subtilis*	*S. aureus*	DPPH radical	Hydroxyl radical
Guava leaf extract	12.1 ± 0.03	13.3 ± 0.01	14.1 ± 0.02	13.6 ± 0.01	47.87 ± 0.07	43.36 ± 0.06
Ampicillin	03.3 ± 0.01	14.7 ± 0.05	13.6 ± 0.01	15.8 ± 0.04	–	–
Control jelly (CJ)	03.1 ± 0.04	07.2 ± 0.01	05.2 ± 0.02	07.7 ± 0.03	26.23 ± 0.04	20.08 ± 0.05
Guava jelly (GJ)	11.4 ± 0.06	12.9 ± 0.01	13.6 ± 0.07	13.1 ± 0.03	42.38 ± 0.06	33.44 ± 0.04

Data is expressed as mean ± standard deviation

Another important criterion that needs to be studied in jelly samples is springiness which is inversely proportional to hardness (Kreungngern and Chaikham 2016). Both the jellies GJ and CJ have exhibited nearly similar springiness i.e., 9.46 ± 0.5 mm and 9.04 ± 0.9 mm respectively. It represents the rate at which the deformed sample regains its initial condition upon removal of the deforming force (Garrido et al. 2014). Springiness depends upon the concentration of the pectin used for jelly preparation (Hamedi et al. 2018). Since the concentration of pectin used in this study is same in both jellies, the springiness results are similar for both the jellies.

Besides the mentioned properties, gumminess of the jellies was calculated as the product of hardness times cohesiveness. Gumminess of GJ (125 ± 9.6 g) is slightly higher than that of CJ (114 ± 5.2 g). The gumminess of a jelly increases with increase in hardness as the energy needed to disintegrate the jelly increases substantially (Mutlu et al. 2018). From the results of texture analysis, it was observed that both the control and the guava extract jellies shared similar properties indicating that, the addition of guava extract does not bring any change in the texture properties of jelly. Moreover, addition of guava leaf extract enriches the jelly with secondary metabolites exhibiting bioactive properties. Secondary metabolites are non-nutritive molecules produced by plants for the sake of their own protection, which were found to possess similar or different effect on humans against various diseases and disorders (Slavin et al. 2017).

The antibacterial activity of guava leaf extract was assessed against four tested bacteria viz., *Bacillus subtilis*, *Proteus vulgaris*, *Staphylococcus aureus* and *Streptococcus mutans*. These bacteria are mostly responsible for causing food spoilage and foodborne diseases which lead towards increased morbidity and mortality (Liu et al. 2017). For instance, *B. subtilis* causes food borne illness by producing heat stable toxin, amylopsin. Similarly, *Staphylococcus* strains cause food poisoning by producing enterotoxins which are responsible for vomiting, diarrhea and in extreme cases it will cause the consumer to collapse. Such unfortunate situations can be overcome in the jelly (GJ) due to the presence of guava leaf extract with antibacterial activity. Least activity was found in *Proteus vulgaris*, due to the presence of thick cell wall made up of lipopolysaccharides that can effectively prevent the passage of leaf extracts (Slavin et al. 2017). Whereas, *Bacillus subtilis*, *Staphylococcus aureus* and *Streptococcus mutans* have a web like cell wall made of peptidoglycan layer, which is comparatively permeable to leaf extracts (Bai et al. 2016). It was reported that nearly 39% of the children consume jams and jellies regularly (Iftikhar et al. 2012). Because of the sticky and sugary nature of jelly, it is predominantly responsible for tooth decay. Bearing this in mind, we attempted to add guava leaf extract possessing antibacterial activity to the jelly which has potential to control the bacterial growth in the mouth. The results shown in the Table 4 authenticates the antibacterial nature of guava leaf extract and the jelly prepared using it as an ingredient.

Antioxidant properties are considered as crucial for food and food supplements these days because free radicals excessively produced inside the body/cell are capable of causing various diseases such as cancer, cardio vascular diseases, neurodegenerative disease etc. (Zhang et al. 2015). In this work, ESR spectroscopy is used to check the activity instead of UV–Vis spectrophotometer because, ESR is considered as one of the most sensitive, direct and effective methods to detect antioxidant activity of biological samples (Kumar et al. 2011). Guava leaf extract was successful in scavenging DPPH* with 47.87% and 43.36% activity against *OH species. This might be due to the presence of compounds like quercetin, myricetin (phenolic compounds) etc., which are known for their therapeutic value with rich antioxidant properties (Chiari-Andreo et al. 2017). Diaz-de-Cerio et al. (2016) has confirmed the same by screening compounds using HPLC as well as elaborated their beneficial effects on various chronic diseases. As antioxidants reduce the oxidative stress and allied diseases, the consumption of GJ can substantially reduce the incidence of these diseases because of its antioxidant properties.

The sensory evaluation of prepared jellies corroborated the acceptability of GJ owing to its appealing colour, smooth texture and good taste (Fig. 3). The prepared product, GJ is having better nutritional value needed for human growth and hence, its consumption adds benefits to the consumers. Moreover, the jellies are consumed by the people of all the ages and thus there is a huge market potential. In order to scale up the jelly production, one should be assured about the abundant availability of the guava leaf as well as conduct optimization studies to obtain high yield of product with uniform quality and texture at every batch of production.

## Data Availability

The data that support the findings of this study are available on request from the corresponding author. The data are not publicly available due to privacy or ethical restrictions.
